# Challenging a host–pathogen paradigm: Susceptibility to chytridiomycosis is decoupled from genetic erosion

**DOI:** 10.1111/jeb.13987

**Published:** 2022-02-28

**Authors:** Donal Smith, David O'Brien, Jeanette Hall, Chris Sergeant, Lola M. Brookes, Xavier A. Harrison, Trenton W. J. Garner, Robert Jehle

**Affiliations:** ^1^ School of Science, Engineering and Environment University of Salford Salford UK; ^2^ Institute of Zoology Zoological Society of London London UK; ^3^ Highland Amphibian and Reptile Project Dingwall UK; ^4^ MRC Centre for Global Infectious Disease Analysis Imperial College School of Public Health London UK; ^5^ 192439 Royal Veterinary College Hatfield UK; ^6^ Centre for Ecology and Conservation University of Exeter Exeter UK

**Keywords:** amphibians, *Batrachochytrium dendrobatidis*, *Bufo bufo*, population fragmentation, SNPs

## Abstract

The putatively positive association between host genetic diversity and the ability to defend against pathogens has long attracted the attention of evolutionary biologists. Chytridiomycosis, a disease caused by the chytrid fungus *Batrachochytrium dendrobatidis* (*Bd*), has emerged in recent decades as a cause of dramatic declines and extinctions across the amphibian clade. *Bd* susceptibility can vary widely across populations of the same species, but the relationship between standing genetic diversity and susceptibility has remained notably underexplored so far. Here, we focus on a putatively *Bd*‐naive system of two mainland and two island populations of the common toad (*Bufo bufo*) at the edge of the species’ range and use controlled infection experiments and dd‐RAD sequencing of >10 000 SNPs across 95 individuals to characterize the role of host population identity, genetic variation and individual body mass in mediating host response to the pathogen. We found strong genetic differentiation between populations and marked variation in their susceptibility to *Bd*. This variation was not, however, governed by isolation‐mediated genetic erosion, and individual heterozygosity was even found to be negatively correlated with survival. Individual survival during infection experiments was strongly positively related to body mass, which itself was unrelated to population of origin or heterozygosity. Our findings underscore the general importance of context‐dependency when assessing the role of host genetic variation for the ability of defence against pathogens.

## Graphical abstract

Chytridiomycosis is responsible for declines or extinctions of hundreds of amphibian species, and therefore regarded as the most devastating vertebrate disease ever recorded. In this paper, we show through experimental infections of common toads (*Bufo bufo*) that susceptibility to chytridiomycosis markedly differs between adjacent populations, but that this difference is not governed by isolation‐mediated erosion of population genetic diversity. At an individual level, susceptibility is further strongly positively related to body mass irrespective of population of origin.

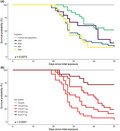

## 1 INTRODUCTION

The positive influence of genetic variation on fitness is central to the field of evolutionary biology and, for example, led to the fundamental notion that costly sexual reproduction is maintained because it increases the level of defence against parasites and disease (Hamilton, 1980; Hamilton et al., 1990; see also, e.g. King et al., 2011). Given that human activities generally erode genetic variation in wild populations while simultaneously promoting the spread of pathogens, the interplay between the standing amount of genetic variation and disease susceptibility is also recognized as important from a conservation point of view (Altizer et al., 2003). For systems in which a potentially lethal pathogen is novel, the neutral genetic diversity of host populations is expected to be positively associated with survival because of the tendency for heterogenous host genotypes to limit pathogen spread (e.g. Ekroth et al., 2019; Gibson & Nguyen, 2020; see also Ostfeld & Keesing, 2012) and the protection conferred by higher genome‐wide heterozygosity at the individual level (e.g. Coltman et al., 1999; Pearman & Garner, 2005; Spielman et al., 2004). Due to previous parasite‐mediated selection and adaptation, systems with a long history of infection are, however, governed by different effects of host gene pools on disease outbreaks than systems that have not been previously severely affected, even leading to situations where selection can lead to decreased functional genetic diversity paralleled with increased disease resistance (e.g. Leeds et al., 2010; Savage & Zamudio, 2016).

Plants and animals are generally unevenly distributed in space. The extent to which they exist as partitioned populations depends on their natural histories as well as the distribution of suitable habitats and broadly increases from the core of a species’ range towards its edge (e.g. Sexton et al., 2009). A particularly high level of natural fragmentation pertains to populations that are distributed across islands, resulting in high levels of local erosion of genetic diversity combined with high degrees of distinctiveness as a function of population size and island connectivity (e.g. Whittaker & Fernández‐Palacios, 2007). Particularly when compared to mainland populations, a given taxon that occupies a series of adjacent islands thus represents an amenable system to study the effects of hierarchical population structure on phenotypic evolution and ecological processes, including the extent to which populations respond to pathogens (Grenfell & Harwood, 1997; Millins et al., 2018; Parratt et al., 2016; Whiteman et al., 2006).

Due to low vagility and life history attributes such as water dependency, amphibians are particularly dispersal limited and often reside in genetically distinct, spatially structured populations (e.g. Hancock & Hedrick, 2018). With marked population declines in all biogeographic regions, they also represent a particularly prominent example of the rapid and severe loss of biodiversity currently underway (Houlahan et al., 2000; Stuart et al., 2004; Wake & Vredenburg, 2008). A main driver for these declines is chytridiomycosis, an emerging infectious disease caused by the fungal pathogen *Batrachochytrium dendrobatidis* (hereafter *Bd*). Chytridiomycosis is regarded as the most devastating vertebrate disease ever recorded and implicated in the declines or extinctions of hundreds of species (O’Hanlon et al., 2018; Scheele et al., 2019).

*Batrachochytrium dendrobatidis* is however not universally destructive, with the severity of disease outbreaks depending on *Bd* strain, amphibian taxonomy and a multitude of environmental factors (for a review see Fisher & Garner, 2020). The impact of infection also varies widely among individuals of the same species, and controlled laboratory experiments have revealed a generally positive association between resistance to *Bd* and body size (Bielby et al., 2015; Carey et al., 2006; Garner et al., 2009; Meurling et al., 2021; Searle et al., 2011). Marked intraspecific variation in *Bd* susceptibility further occurs between distinct populations, suggesting that innate genetic factors are acting (Tobler & Schmidt, 2011; Luquet et al., 2012; Bataille et al., 2015; Bradley et al., 2015; see also Palomar et al., 2016). After an outbreak has occurred, field surveys linking *Bd* prevalence with host neutral genetic variability have yielded mixed findings, with positive (Addis et al., 2015; Horner et al., 2017), negative (Savage et al., 2015) and neutral (Wagner et al., 2017) associations all reported. Given *Bd's* impact as a pathogen when it invades an area for the first time, the role played by levels of standing genetic variation prior to exposure in predicting the immediate responses of host populations is, therefore, a critical question that has, however, so far remained underexplored.

Despite recent declines, the common toad (*Bufo bufo*) remains one of the most widespread amphibians in northern Europe, where fragmented population structures on islands and adjacent coastlines have been documented (O’Brien et al., 2021; Roth & Jehle, 2016; Seppä & Laurila, 1999; Tuncay et al., 2018). As a result of its intermediate susceptibility to *Bd* (e.g. Kärvemo et al., 2019), *B. bufo* has also served as a model species to unravel key aspects of chytridiomycosis, including the discovery of the highly virulent *Bd*GPL lineage (Farrer et al., 2011; Fisher et al., 2009) and the protective role played by body mass and development to reduce *Bd* susceptibility (Bielby et al., 2015; Garner et al., 2009, 2011; Meurling et al., 2021). In the present study, we sample *B. bufo* individuals from mainland and inshore islands at the edge of the species’ range and characterize these populations using a high‐density panel of de novo assembled SNP markers. Using controlled infection experiments, we investigate whether population‐level genetic diversity or individual multi‐locus heterozygosity affect the capacity to survive exposure to *Bd*, taking body mass as a known phenotypic determinant of *Bd* susceptibility into account.

## 2 MATERIALS AND METHODS

### 2.1 Sample collection and animal husbandry

**FIGURE 1:**
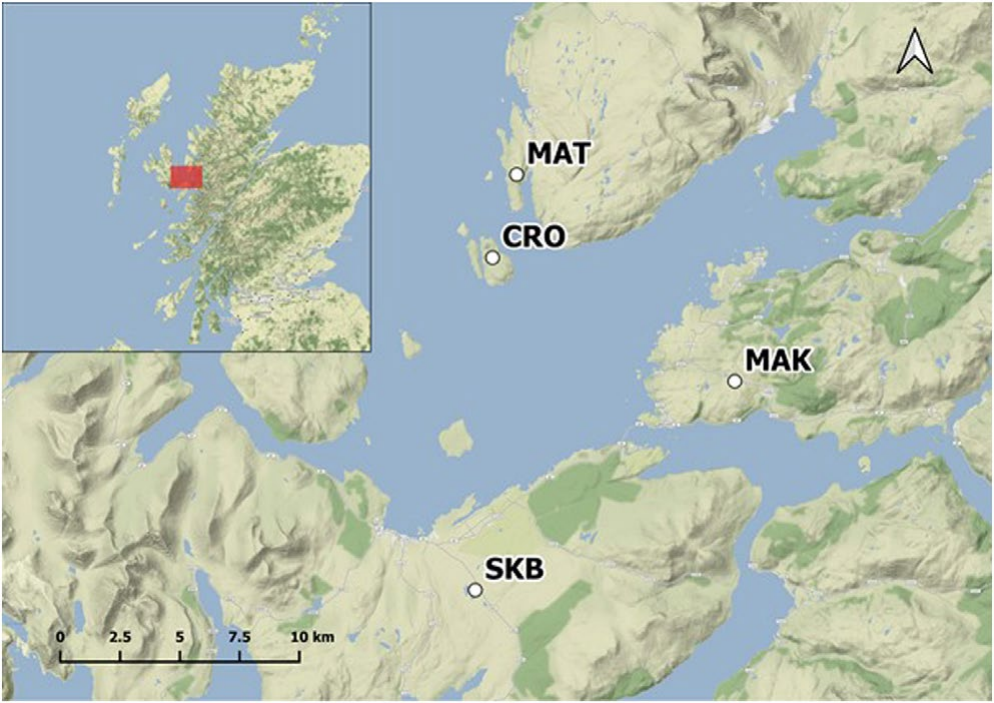
Study area in western Scotland, with locations and names of sampling sites. CRO, Crowlin; MAK, Mainland, Kyle of Lochalsh; MAT, Mainland, Toscaig; SKB, Skye, Broadford

Our study was based on four *B. bufo* populations in western Scotland (Figure 1), which were presumed to be *Bd*‐naïve as a national survey revealed that the closest site with a positive record was over 280 km south of the study area (Cunningham & Minting, 2008). Two populations (MAK, MAT) were situated on the mainland, one population (SKB) was situated on the large (1656 km^2^) island of Skye separated from the mainland by about 300 m, and one population (CRO) was located on the small island of Crowlin (approximately 1 km^2^ in area and 1.5 km from the mainland), isolated by sea at least since the last period of glacial activity ended approximately 9500 years ago (Lambeck, 1993). Geographic distances between the populations ranged from approximately 3 km (MAT‐CRO) to approximately 12 km (MAT‐SKB, Figure 1).

*Bufo bufo* is an explosive synchronous breeder, and all animals used in the experiment were equivalent in age, being young‐of‐the‐year collected as spawn over a three‐day period in March 2016. Nineteen clutches (egg strings, 3–6 per population) were sampled by collecting approximately 10 cm sections from each of them. Eggs were transported to the Institute of Zoology, Zoological Society of London, where they were hatched in UK Home Office‐approved outdoor animal facilities and housed separately in 9 L polypropylene boxes (Really Useful Products LTD) containing dechlorinated, aged tap water and an air‐driven sponge filter system with cover and vegetation. Partial (approximately 20%) water changes, as well as feeding using crushed Tetra Tabimin^®^ tablets, were carried out every two days. After metamorphosis, individuals were housed by clutch in 360 × 210 × 160 mm terrestrial tanks (Faunarium, ExoTerra^®^) lined with moist paper towelling and containing a cover object and water container. All individuals were fed hatchling crickets *ad libitum* and were subject to daily health checks, and biosecurity and sanitation were maintained throughout animal raising and experimental procedures.

### 2.2 *Bd* exposure trials

Experimental procedures took place in a temperature‐controlled room at 18°C with a 12:12‐h light:dark cycle. Ninety days after the start of metamorphosis, toadlets were weighed to the nearest 0.001 g and transferred to individual housing in 0.7 L polypropylene boxes (Really Useful Products LTD) lined with a moist paper towel and containing a cover object. Following a 2‐week period of acclimatization, 202 toadlets were randomly allocated into one of two experimental treatments: (I) exposure to an active *Bd* culture or (II) a control group exposed to culture media alone. Treatments broadly followed Garner et al. (2009, 2011). Each toadlet was placed into an individual Petri dish filled with 30 ml of aged tap water and 450 μl of culture media for four hours. In order to ensure a level of exposure sufficient to cause mortality, nine such treatments were carried out across 21 days. All individuals in the exposed group received the same dosage of the *Bd* isolate UK CORN'12 3.1, part of the hypervirulent global panzootic lineage *Bd*GPL (Farrer et al., 2011). Dosages were calculated by counting live spores using a haemocytometer and varied between 40 500 and 382 500 zoospores depending on session. The experiment proceeded for 50 days after the first exposure (i.e. a 21‐day period of inoculations followed by a further 29 days of daily monitoring of mortality). If a toadlet reached a humane end point (i.e. was unresponsive or was unable to support its own weight), it was euthanised by licenced personnel in accordance with the Animal (Scientific Procedures) Act 1986 using the non‐schedule 1 method of immersion in buffered tricaine methanesulphonate (MS222) followed by fixation in 70% ethanol. All animals surviving to the end of the experiment were euthanised and fixed as described above.

### 2.3 Genotyping and *Bd* detection

Toe clips from the hind foot of all experimental individuals were sampled for *Bd* screening after death, using the qPCR assay protocol described in Boyle et al. (2004). DNA was extracted using Prepman™ Ultra (Applied Biosystems), and samples were analysed in duplicate using a TaqMan^®^ qPCR assay (Applied Biosystems) with four dilution standards representing 100, 10, 1 and 0.1 *Bd* zoospore genomic equivalents (*Bd*GE), and a negative control. In the case where only one replicate amplified, that sample was reanalysed. This occurred with three samples, which were negative in subsequent analyses.

DNA for genotyping was extracted from hind leg muscle of toadlets using a Qiagen DNEasy extraction kit following the manufacturer's protocol (Qiagen). DNA concentration was assessed by fluorometry using a Qubit 3.0 (Thermo Fisher Scientific) and standardized to 20 ng/µl. The set of 101 exposed individuals (excluding the controls) was reduced to 95 by randomly removing six individuals from the best‐represented population (MAK). The resulting panel of samples constituted 15 individuals from CRO, 31 from MAK, 27 from MAT and 22 from SKB. Preparation of dd‐RAD libraries and next‐generation DNA sequencing were performed by Floragenex following the protocol of Truong et al. (2012). In brief, DNA was double digested with a combination of rare and frequent cutting endonucleases (*Pst*I and *Mse*I, respectively), followed by ligation with adaptors with individual indices. 1 × 100bp single‐end sequencing was performed on the resulting PCR‐generated library using an Illumina HiSeq 4000.

Once obtained from Floragenex, a de novo catalogue of SNP loci was constructed using stacks 2.0 (Catchen et al., 2013), given that the *B. bufo* genome (Streicher & Darwin Tree of Life Consortium, 2021) was not yet available at the time of the analyses. The raw sequences were filtered and demultiplexed using the process_radtags pipeline. Reads with an uncalled base were discarded, as were reads containing a 15 bp window in which the average quality dropped below a phred score of 10 (i.e. a 90% probability of being correct). Barcodes and RAD‐tags containing one mismatch to an expected sequence were retained. To identify an optimal set of parameter values in stacks, we followed procedures described in Paris et al. (2017) and Rochette and Catchen (2017). Based on these considerations, a value of 4 was chosen for both the *M* and *n* parameters, which control the number of mismatches permitted between two alleles of an individual heterozygote locus and two alleles in a locus across a population, respectively.

### 2.4 Data analyses

Observed (*H*_o_) and expected (*H*_e_) heterozygosities for each population were calculated using the *R* package adegenet (Jombart, 2008). Deviations from Hardy–Weinberg equilibrium and pairwise *F*_ST_ values were calculated using the software genepop 4.4 (Rousset, 2008; input files were generated from vcf files using pgdspider 2.1.1.5, Lischer & Excoffier, 2012). Individual heterozygosities were calculated using the *R* package inbreedr (R Core Team, 2018; Stoffel et al., 2016).

To identify genetic clusters of individuals, we also performed a Discriminant Analysis of Principal Components (DAPC, Jombart et al., 2010) in adegenet. Clusters were inferred using sequential *K*‐means clustering. Runs were performed sequentially using increasing values of *K* (number of clusters) without providing prior information on the populations of origin and compared using the Bayesian information criterion (BIC), with the preferred value of *K* defined by the elbow of the curve of BIC values when graphed against increasing values of *K*.

The relationships between the four study populations were also inferred using snapp 2.6 (Bryant et al., 2012) implemented in beast 2.5 (Bouckaert et al., 2019). snapp infers population trees from unlinked markers by implementing a coalescent model with an algorithm which considers all possible gene trees. To reduce computational run times, we selected five random individuals from different clutches for each population. Backwards (*u*) and forwards (*v*) mutation rates (expected mutations per site per generation) were estimated from the data in snapp, with the birth rate (*λ*) of the Yule prior (indicating the rate at which populations diverge from one another) equalling the number of samples used. We used a chain length of 10 000 000 generations sampling every 1000 trees and visualized the resulting tree using densitree (Bouckaert, 2010) as implemented in beast. An absolute dating of nodes derived from snapp is difficult to achieve without detailed biogeographic information and can be further biased due to gene flow, non‐dependence of loci as well as selection (e.g. Barratt et al., 2018; Stange et al., 2018). We, therefore, refrained from a time calibration of the obtained tree.

To evaluate the factors influencing survival in response to *Bd* exposure, Cox Proportional Hazard (CPH) analyses were performed incorporating the explanatory variables population of origin, individual mean heterozygosity and pre‐exposure body mass (cg). Each variable was tested to confirm adherence to model assumptions. These tests were carried out using the survival package version 2.38 (Therneau, 2015) in *R* 3.5.0 (R Core Team, 2018). To further illustrate the impact of mass on survival, individuals were divided into quartiles based on mass, and Kaplan–Meier survival curves were plotted for these groupings as well as for populations.

## 3 RESULTS

### 3.1 Genetic structure of populations

The final filtered SNP data set contained a total of 10 884 biallelic loci. As expected, the mainland populations were characterized by higher levels of heterozygosity (MAK: 0.316, MAT: 0.333) compared with the island populations (CRO: 0.263, SKB: 0.260), with no significant deviations from Hardy–Weinberg equilibria in all cases (*p* > 0.05). Pairwise *F*_st_ values between populations ranged from 0.12 between the two mainland populations (MAK‐MAT) to 0.32 between the two island populations (CRO‐SKB); the remaining pairwise comparisons had intermediate *F*_st_ values of 0.19 (CRO‐MAT) and 0.20 (CRO‐MAK and MAK‐SKB).

**FIGURE 2:**
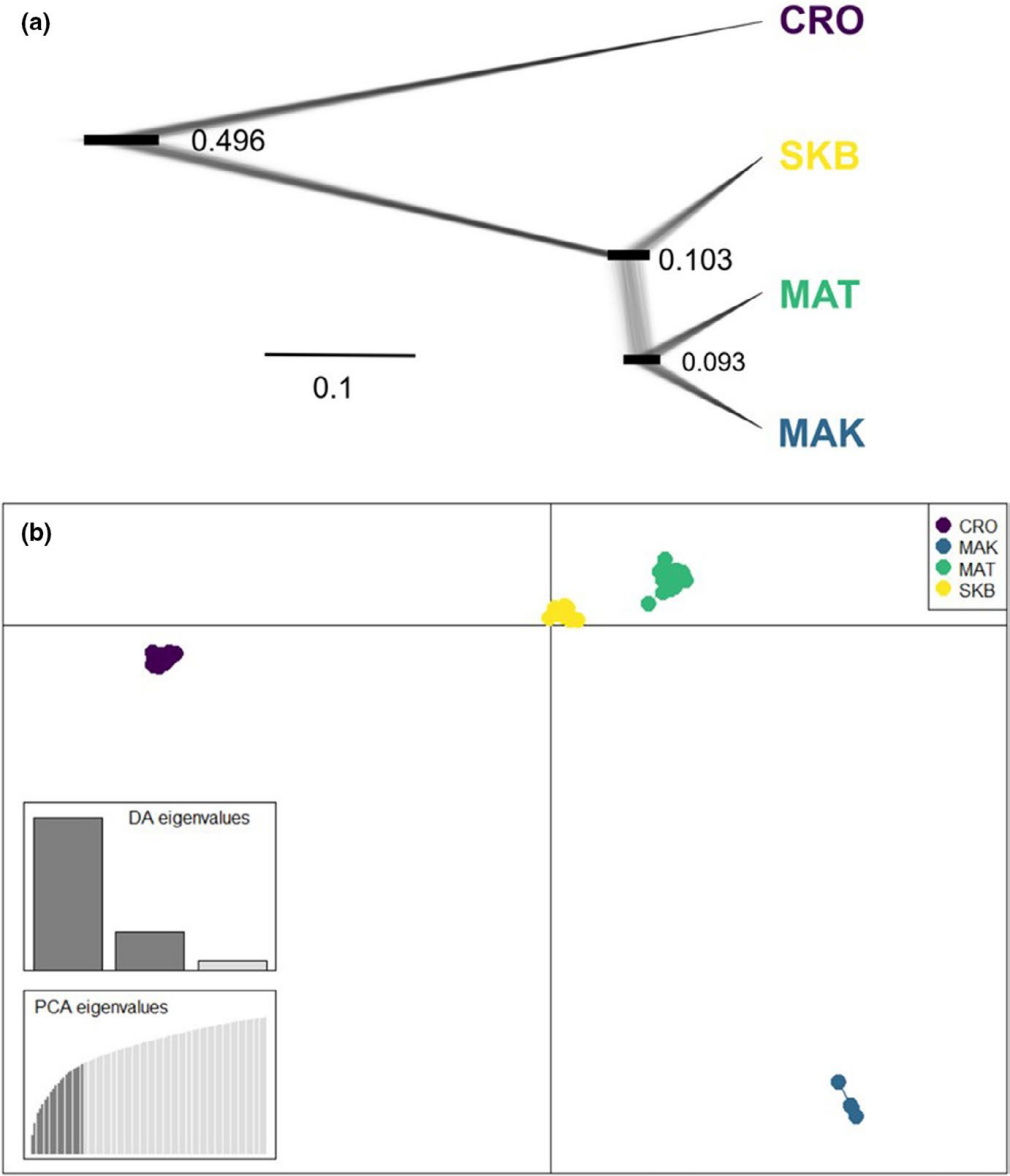
Genetic structure of the four study populations. (a) SNP‐based phylogenomic tree as inferred by the snapp algorithm; node labels denote relative node ages, with bars showing 95% highest posterior density intervals. (b) Scatterplot of the first two principal components of DAPC analysis. Each individual is coloured according to its population of origin

The *K*‐means algorithm implemented in adegenet identified four genetic clusters, assigning all individuals into groups which corresponded with their populations of origin. The DAPC plot shows that individuals are tightly clustered within their respective populations, which are well separated along the first PC axis alone (Figure 2b). The phylogenomic algorithm implemented in snapp confirms the distinctiveness of the island population CRO in accordance with the geographic setting; SKB on the large island of Skye is characterized by a similar putative split to that of the two mainland populations MAK and MAT, which are separated by overland distances exceeding 20 km (Figure 2a).

### 3.2 Individual‐ and population‐level response to experimental Bd exposure

**FIGURE 3:**
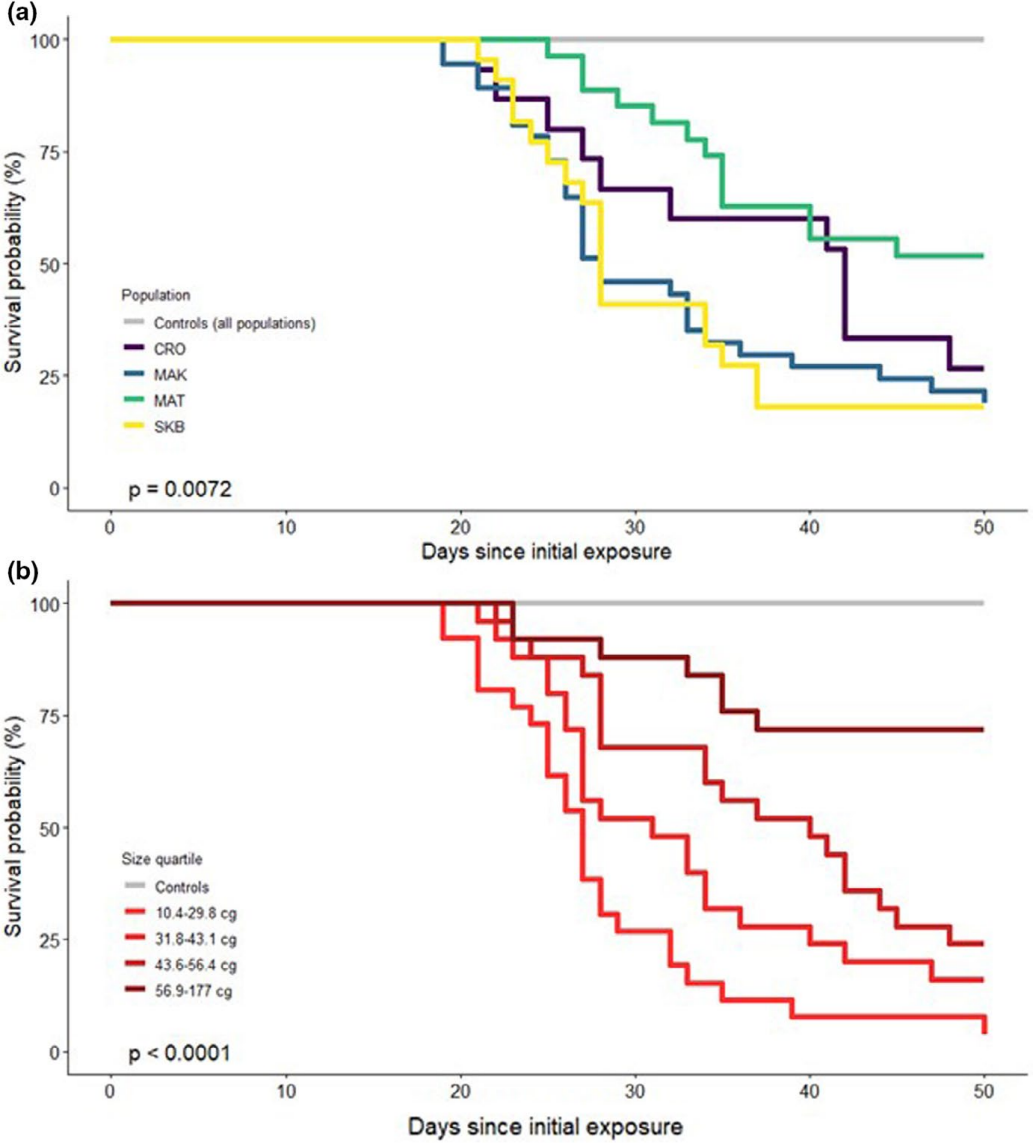
Kaplan–Meier survival curves comparing the survival of (a) four *Bd* challenged populations and controls, and (b) mass quartile groupings of *Bufo bufo* individuals experimentally exposed to *Bd*. All control animals survived (grey lines)

Within 50 days of the initial exposure, 72 of 101 (71%) toadlets exposed to *Bd* died, whereas all 101 control animals survived. All experimental mortalities and 35% (10/29) of exposed animals surviving to the end of the experiment were recorded as infected, whereas no *Bd* was detected in the unexposed control group. Infection prevalence for exposed individuals at point of death was 70% for MAT, 80% for CRO and 86% for MAK and SKB. Survival was 18% (4/22) for SKB, 19% (7/37) for MAK, 27% (4/15) for CRO and 52% (14/27) for MAT (Figure 3a, with median survival times of 28, 28, 42 and >50 days, respectively) and significantly differed between populations (logrank test = 12.6, d.f. = 3, *p* = 0.007). When compared to the population in which survival was highest (MAT), population membership significantly determined mortality when controlling for the effect of body mass (CPH coefficients ranging from 0.926 to 1.046, see Table 1); the membership of clutch, on the contrary, did not show a significant effect (at however low sample sizes per clutch, data not shown).

**TABLE 1:** Cox Proportional Hazard (CPH) model for survival of *Bufo bufo* toadlets exposed to *Bd*. Model included mass (per cg), individual multi‐locus SNP heterozygosity (%) and population of origin (for which CPH coefficients are with reference to the population with the highest survival, MAT)

	Coefficient	SE	*z*	Hazard ratio (95% CI)	*p*
Mass (cg)	−0.063	0.010	−6.303	0.940 (0.922–0.958)	<0.001
SNP MLH (%)	0.324	0.112	2.904	1.383 (1.111–1.721)	<0.01
Populations (relative to MAT)					
CRO	2.714	0.777	3.491	15.086 (3.288–69.221)	<0.001
MAK	0.985	0.395	2.491	2.680 (1.234–5.82)	0.013
SKB	3.636	0.776	4.684	37.929 (8.284–173.653)	<0.001

Abbreviation: MLH, multi‐locus heterozygosity.

**FIGURE 4:**
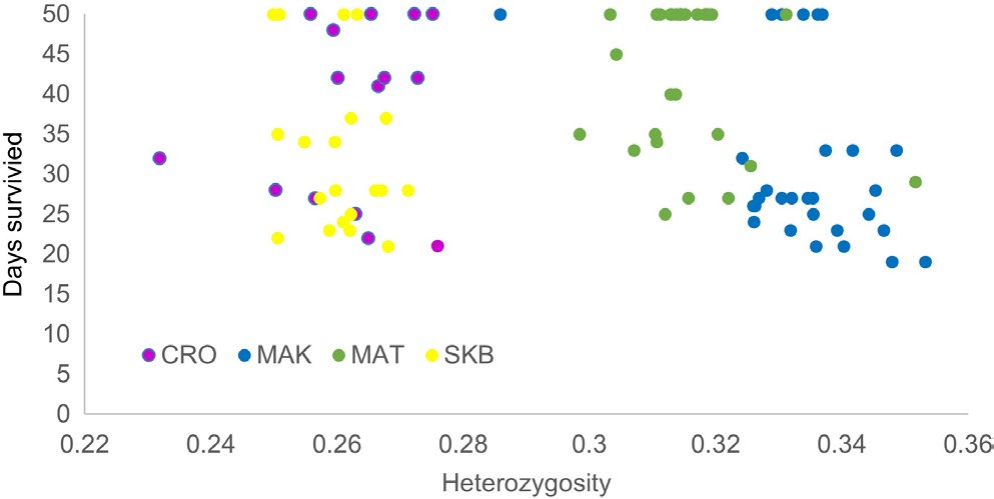
Relationship between individual heterozygosity and disease trial performance (days survived) of *Bufo bufo* individuals exposed to *Bd*. CRO and SKB are island populations, and MAK and MAT are mainland populations

Individual mass of exposed individuals did not vary between populations (ANOVA, *F*(3, 97) = 1.211, *p* = 0.31), but had a highly significant effect on survival during trials, with an additional 1 cg of mass leading to an approximately 6% decrease in mortality risk per day when controlling for population (CPH coefficient in combined model = −0.063, *p* < 0.001, Table 1). This was also reflected in the significant difference in survival times between mass quartiles (logrank test = 39.4, d.f. = 3, *p* < 0.001. Figure 3b). SNP heterozygosity was not associated with mass (either overall or within population, Spearman rank correlation *p*‐values >0.10 in all cases), nor was it found to be associated with survival when evaluated independently in a CPH (see also Figure 4). In the combined model (Table 1), however, SNP multi‐locus heterozygosity was found to have a significant negative relationship with survival when accounting for population and mass.

## 4 DISCUSSION

The present study combined controlled infection trials with high‐throughput genotyping to investigate whether host genome‐wide genetic variation is linked to disease susceptibility in the chytrid‐amphibian system. Our results confirm that island populations were subject to elevated levels of genetic erosion compared with populations on the mainland and demonstrate that the study populations responded differentially to pathogen exposure. We also replicate the observation that larger body size protects against the negative effects of *Bd* exposure. However, contrary to the fundamental paradigm that host genetic diversity impedes pathogen success (Coltman et al., 1999; Pearman & Garner, 2005; Spielman et al., 2004), the observed variation in survival was not driven by isolation‐mediated population genetic erosion and even showed a negative association with individual heterozygosity. Our study underscores the importance of context‐dependency when assessing the interplay between host genetic variation and disease challenges and addresses open questions on the role of population demographic history in mediating the threat posed by contact with a devastating novel pathogen such as *Bd*. It also disentangles the strong effect of body size on *Bd* susceptibility from the complexities of intrinsic genetic effects.

Our finding of a pronounced spatial genetic structure aligns with other studies on northern European *B. bufo* populations (e.g. Brede & Beebee, 2004; Thörn et al., 2021), with the high‐density panel of SNPs unequivocally demonstrating a reduced level of heterozygosity in island populations compared with the mainland, an effect which was less apparent in previous studies based on fewer loci (Roth & Jehle, 2016; Seppä & Laurila, 1999). The early putative split of the population on the small island (CRO) confirms evidence from microsatellites and mtDNA that supports its natural origin likely through rafting events during the melting of glaciers as opposed to a recent human introduction (O’Brien et al., 2021).

The ability to carry the costs incurred by an infection intuitively depends on the phenotype. While a positive relationship between body mass and resistance or resilience to disease is not universally present across all host‐parasite systems (for a meta‐analysis see Sánchez et al., 2018), our finding of increased survival in larger individuals is consistent with previous *Bd* exposure experiments conducted with *B. bufo* (Bielby et al., 2015; Garner et al., 2009, 2011; Meurling et al., 2021) and a range of other anuran species (Bradley et al., 2015; Carey et al., 2006; Kriger et al., 2006; Searle et al., 2011; Tobler & Schmidt, 2011). *Bd* infection is confined to the epidermis, and smaller individuals exhibit higher relative rates of energetically costly skin sloughing and ion loss than their larger counterparts (Wu et al., 2018). Despite an initially smaller potential area of exposure, they also may generally be more vulnerable because both surface area and metabolic rate scale allometrically with mass (Klein et al., 2016; White et al., 2006). Our SNP data revealed that the observed individual differences in mass are not mediated by an effect of heterozygosity on attained size. This finding contrasts a previous study which demonstrated a link between genetic variation and developmental rates in *B. bufo* populations suffering from urbanization (Hitchings & Beebee, 1998; see also Rowe et al., 1999; Lesbarrères et al., 2007 for similar findings in other anuran species), suggesting that the natural, long‐term genetic erosion of island populations might not result in the same adverse effects as more recent fragmentation caused by human habitat conversion. Milder winters may also drive smaller female body size in *B. bufo* (Coles et al., 2019; Reading, 2007), coupled with a projected expansion of the range of *Bd* in temperate zones of the Northern Hemisphere associated with a warming climate (Xie et al., 2016) suggesting that the role played by host body size in amphibian chytridiomycosis may warrant greater attention.

Our study used controlled infection experiments to characterize the role of host genetic diversity in mediating variation in susceptibility to chytridiomycosis among natural populations. We show that, despite significant population‐level variation in *Bd*‐driven mortality in our system, isolated and less diverse populations are not intrinsically more vulnerable. It is possible that, while genetic variation is lower in our island populations, it is not sufficiently reduced to result in negative heterosis compared with the mainland populations. This, however, still fails to explain why population of origin played an important role in disease susceptibility. MAK, a mainland population with the highest level of observed heterozygosity, showed lower survival compared with the neighbouring mainland population (MAT) and even the isolated island population CRO. Our unexpected finding of a negative association between survival and heterozygosity could be caused by advantageous additive genetic variants, whose homozygote state is reflected in low genome‐wide levels of genetic variation. While a GWAS approach proved unsuited due to sample‐size limitations (See Table S1), future studies could also more specifically target disease‐adaptive genes and their expression patterns for our study populations (MHC: e.g. Cortazar‐Chinarro et al., 2019; Kosch et al., 2019; Trujillo et al., 2021; see also Gauberg et al., 2019 for gene expression effects on tight junction proteins). Ultimately, however, the pathways underpinning disease can be highly complex, consisting of multiple steps and influenced by the interactions of a large number of potentially pleiotropic genes with small individual effect sizes (Fenton et al., 2012; Manolio et al., 2009). Originating from separate founder events and with a high degree of divergence (see also O’Brien et al., 2021), the populations in our system are expected to lead to sets of study individuals which are partitioned by their distinct genetic architectures. From this view, it appears plausible that the high degree of divergence between our populations alone could have caused the distinct, seemingly idiosyncratic responses to pathogen exposure irrespective of the effects of heterozygosity or the potential contribution of a small number of loci which are linked to disease resistance.

In a disease outbreak, the fates of affected populations are influenced by a multitude of additional factors. The monoculture effect, for example, predicts that genetic homogeneity of hosts facilitates the spread of pathogens, leading to worse outcomes in less diverse populations (Ekroth et al., 2019; Gibson, 2021; Gibson & Nguyen, 2020). Conversely, the very phenomenon that gives rise to lower diversity, demographic isolation, should act to inhibit the arrival of novel pathogens (see e.g. Millins et al., 2018 for a mammalian host‐parasite system). The positive influence of population‐level diversity may also manifest over time, as successive generations of coevolution select for more resistant genotypes (see e.g. Savage & Zamudio, 2016). Nevertheless, given *Bd's* ability to cause abrupt declines and extinctions immediately upon its arrival in amphibian communities, a population's capacity to exert its evolutionary potential may well be dependent on its ability to survive the first contact with the pathogen. It is in this respect that our findings caution against simplistic expectations about the comparative vulnerability of genetically depauperate populations. The genetic characteristics of host populations clearly play a role in shaping response to *Bd*, but identifying in advance those most at risk remains a challenge.

## CONFLICT OF INTEREST

The authors have no conflict of interest to declare.

## AUTHOR'S CONTRIBUTIONS

DO’B and JH provided fieldwork expertise; DS, RJ, DO’B and JH conducted the fieldwork; DS, CS and LMB performed the animal husbandry work; DS performed the experimental infections; DS, TWJG, XAH and RJ conceived the study, TWJG, XAH and RJ coordinated the study; DS and RJ analysed the data and wrote the manuscript, with input from all authors.

### PEER REVIEW

The peer review history for this article is available at https://publons.com/publon/10.1111/jeb.13987.

## Supporting information

Table S1

## Data Availability

The data that support the findings of this study are openly available in Dryad at http://doi.org/10.5061/dryad.9s4mw6mjb
