# Supplementary material for: Challenging a host–pathogen paradigm: Susceptibility to chytridiomycosis is decoupled from genetic erosion
Source: J Evol Biol. 2022 Feb 28;35(4):589–98. doi: 10.1111/jeb.13987 (PMC9306973; doi:10.1111/jeb.13987)
Supplement: Supplementary file 1 — Table S1 [file JEB-35-589-s001.docx]

Supplement 1: Clutch-level data (sample sizes and survival rates) for *Bufo bufo* toadlets exposed to *Bd.*

| **Population** | **Clutch** | **Number exposed** | **Surviving** | **Clutch survival rate** | **Total exposed** | **Total surviving** | **Population survival rate** |
| --- | --- | --- | --- | --- | --- | --- | --- |
| CRO | CRO1 | 5 | 1 | 0.20 | 15 | 4 | 0.27 |
|  | CRO2 | 3 | 2 | 0.67 |  |  |  |
|  | CRO5 | 7 | 1 | 0.14 |  |  |  |
| MAK | MAK1 | 8 | 1 | 0.13 | 37 | 7 | 0.19 |
|  | MAK2 | 7 | 4 | 0.57 |  |  |  |
|  | MAK3 | 9 | 1 | 0.11 |  |  |  |
|  | MAK4 | 7 | 0 | 0.00 |  |  |  |
|  | MAK5 | 4 | 1 | 0.25 |  |  |  |
|  | MAK6 | 2 | 0 | 0.00 |  |  |  |
| MAT | MAT1 | 6 | 3 | 0.50 | 27 | 14 | 0.52 |
|  | MAT2 | 5 | 3 | 0.60 |  |  |  |
|  | MAT3 | 4 | 2 | 0.50 |  |  |  |
|  | MAT4 | 1 | 1 | 1.00 |  |  |  |
|  | MAT5 | 2 | 1 | 0.50 |  |  |  |
|  | MAT6 | 9 | 4 | 0.44 |  |  |  |
| SKB | SKB2 | 6 | 1 | 0.17 | 22 | 4 | 0.18 |
|  | SKB4 | 5 | 1 | 0.20 |  |  |  |
|  | SKB5 | 3 | 1 | 0.33 |  |  |  |
|  | SKB7 | 8 | 1 | 0.13 |  |  |  |
